# The Role of the Arginine in the Conserved N-Terminal Domain RLFDQxFG Motif of Human Small Heat Shock Proteins HspB1, HspB4, HspB5, HspB6, and HspB8

**DOI:** 10.3390/ijms19072112

**Published:** 2018-07-20

**Authors:** Vladislav M. Shatov, Stephen D. Weeks, Sergei V. Strelkov, Nikolai B. Gusev

**Affiliations:** 1 Department of Biochemistry, School of Biology, Moscow State University, Moscow 119991, Russia; shatovm@inbox.ru; 2 Laboratory of Biocrystallography, Department of Pharmaceutical and Pharmacological Sciences, KU Leuven, Leuven 3000, Belgium; stephen.weeks@kuleuven.be (S.D.W.); sergei.strelkov@kuleuven.be (S.V.S.)

**Keywords:** small heat shock proteins, oligomer structure, chaperone-like activity, disease-related mutations

## Abstract

Although the N-terminal domain of vertebrate small heat shock proteins (sHsp) is poorly conserved, it contains a core motif preserved in many members of the sHsp family. The role of this RLFDQxFG motif remains elusive. We analyzed the specific role of the first arginine residue of this conserved octet sequence in five human sHsps (HspB1, HspB4, HspB5, HspB6, and HspB8). Substitution of this arginine with an alanine induced changes in thermal stability and/or intrinsic fluorescence of the related HspB1 and HspB8, but yielded only modest changes in the same biophysical properties of HspB4, HspB5, and HspB6 which together belong to another clade of vertebrate sHsps. Removal of the positively charged Arg side chain resulted in destabilization of the large oligomers of HspB1 and formation of smaller size oligomers of HspB5. The mutation induced only minor changes in the structure of HspB4 and HspB6. In contrast, the mutation in HspB8 was accompanied by shifting the equilibrium from dimers towards the formation of larger oligomers. We conclude that the RLFDQxFG motif plays distinct roles in the structure of several sHsp orthologs. This role correlates with the evolutionary relationship of the respective sHsps, but ultimately, it reflects the sequence context of this motif.

## 1. Introduction

Small heat shock proteins (sHsps) form a large family of chaperones expressed in all kingdoms of life including viruses, bacteria, fungi, plants, and animals [1,2]. Monomers of sHsps have a molecular weight ranging from 13 up to 43 kDa and contain a conserved α-crystallin domain (ACD) consisting of 80–100 residues that form a compact β-sandwich [3,4,5]. The ACD is flanked by a poorly ordered N-terminal domain (NTD) and a comparatively shorter C-terminal domain (CTD). The latter can be further divided into the conserved (I/V/L)-X-(I/V/L) tripeptide (IXI) or C-terminal anchoring motif (CAM) [6], and the highly flexible and polar C-terminal extension (CTE) [5,7,8]. The ACD is responsible for formation of sHsp dimers, whereas the CTD mediates the interdimer interaction [7,8,9]. The sHsp homo- or heterodimers are the building blocks of larger oligomers [10,11]. In addition, sHsp monomers were also shown to participate in the dynamic association of larger homo- and heterooligomers [8]. Oligomerization plays an important role in the regulation of chaperone-like activity and interaction of sHsp with their protein targets [12,13]. 

The NTD is involved in higher order assembly of sHsps and the formation of heterooligomers. The NTD is enriched in Pro and Arg residues, is highly susceptible to proteolysis [14], and often contains multiple phosphorylation sites [15,16,17,18]. Most of the NTD is believed to be intrinsically disordered [19]. The NTD contains little secondary structure, although short α-helices and β-structural elements were observed in plant and yeast sHsps [20,21]. While the high degree of disorder makes it difficult to investigate the structural organization of the NTD, various approaches were used to analyze its functional role. Deletion of certain parts of the NTD [22,23,24,25], domain swapping [26,27], replacement of certain residues [28], and production of phosphomimicking mutants [12,16] have yielded a complex yet intriguing picture. At the same time, large deletions or domain swapping can induce dramatic changes in the overall sHsp assembly, and thus complicate unequivocal interpretation of the results. In addition, these experiments were mostly performed on single members of the sHsp family, predominantly on αA- or αB-crystallins (HspB4 and HspB5, respectively) [16,25,28] or on HspB6 [24,27], while extrapolation to other members of the sHsp family remains problematic.

**Figure 1:**
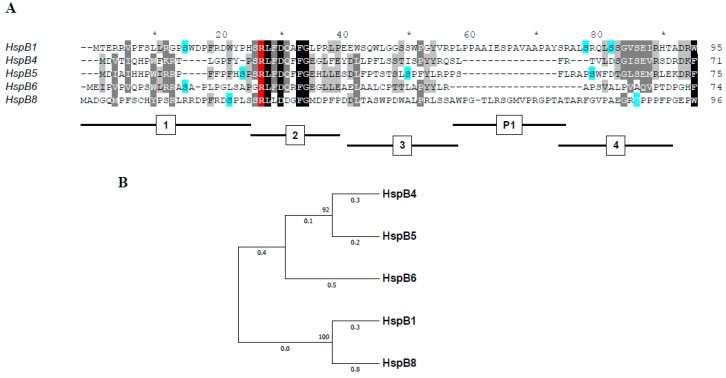
(**A**) Alignment of the N-terminal domains of five human small heat shock proteins. Conservative residues are marked by shades of grey, phosphorylation sites are marked blue, conservative Arg residue is marked red. The subdivision of the NTD is marked below the alignment. (**B**) Phylogeny of five human small heat shock proteins. The following sequences from Uniprot: P04792 (HspB1), P02489 (HspB4), P02511 (HspB5), O14558 (HspB6), Q9UJY1 (HspB8) were used. Multiple alignment was generated by Tcoffee (available online: http://tcoffee.crg.cat/). The tree was inferred by using the Maximum Likelihood method based on the JTT matrix-based model with MEGA7. The tree is drawn to scale, with branch lengths measured in the number of substitutions per site (next to the branches). All positions containing gaps and missing data were eliminated.

The NTD of vertebrate sHSPs can be provisionally divided into four regions [29] (Figure 1A). The first region, corresponding to the immediate N-terminal residues, is 20 amino acids long in the α-crystallins (HspB4 and HspB5) and ~25 residues in HspB1, HspB6, and HspB8. This region is enriched in hydrophobic and aromatic residues and contains a number of phosphorylation sites. The second region, comprising a further 12–15 residues, contains the RLFDQxFG motif that is highly conserved in most vertebrate sHsps except for HspB3 and HspB7, and modified in the case of HspB9 and HspB10 (Figure 1A). The third region, containing residues 42–58 of HspB1 and the corresponding residues of four other sHsps, is poorly conserved and again contains a number of hydrophobic and aromatic residues. HspB1 and HspB8 also contain the so-called P1 region which is lacking in HspB4, HspB5, and HpsB6 (Figure 1A). Dependent on the way the amino acid sequence alignment is performed, the fourth region (or probably extension of the third region) appears to include residues 75–90 of HspB1 or the corresponding residues in other sHsps (Figure 1A). 

The conserved RLFDQxFG motif was shown to play an important role in assembly and heterooligomerization [24,27]. In particular, the first Arg residue of this motif is preserved in sHsps in humans and many other vertebrates, suggesting that this residue may be important for maintaining various structural and functional properties. Here we aimed at a systematic examination of the role of this highly conserved Arg residue in HspB1, HspB4, HspB5, HspB6, and HspB8. After replacing this Arg by Ala in all five proteins, we have analyzed the effect of this replacement on quaternary structure and further biophysical properties. Interestingly, the largest impact of the mutation has been observed in human HspB8 which is particularly important due to disease-related mutations that were discovered in this protein and that correlate with several neurodegenerative and neuromuscular diseases [30,31,32].

## 2. Results

### 2.1. Spectral Properties and Thermal Stability of sHsps and Their Point Mutants

**Figure 2:**
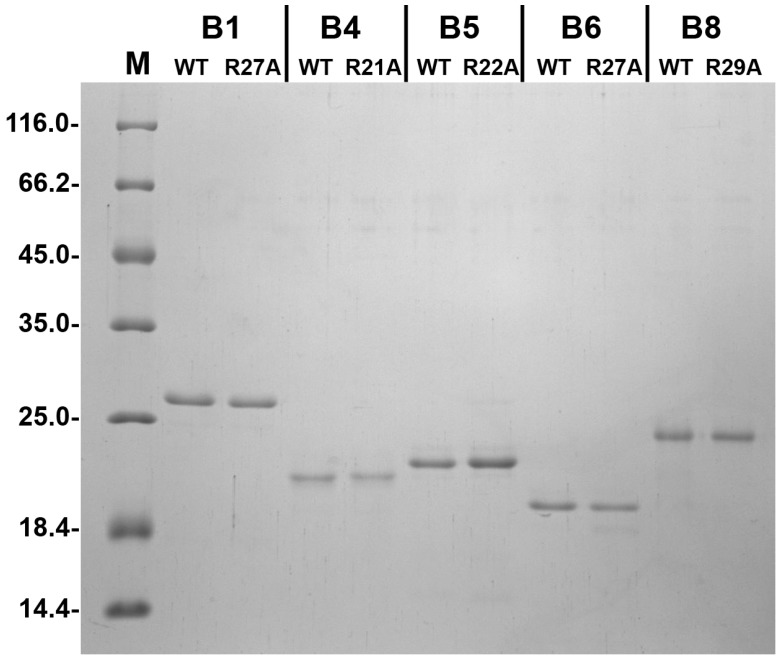
SDS electrophoresis of five human sHsp and their Ala mutants. Equal quantities (~1 µg) of each protein were loaded on each track. The position of markers (M) and their molecular weight (in kDa) are marked by dashes.

**Figure 3:**
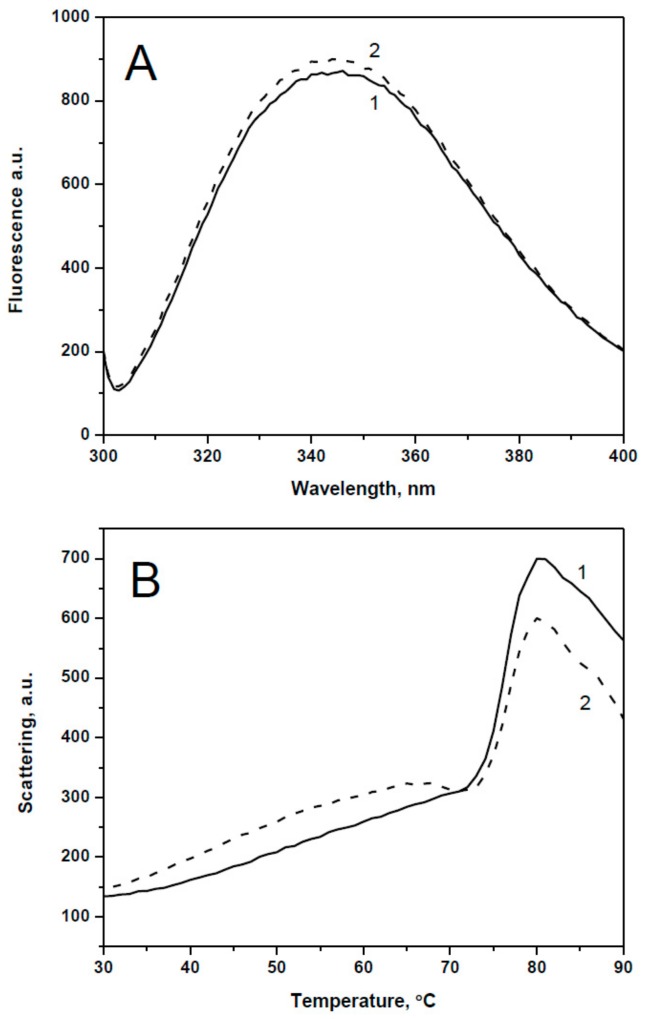
(**A**) Fluorescence spectra of the wild type HspB1 (1) and its R27A mutant (2) excited at 295 nm (slit width 5 nm) and recorded in the range of 300-400 nm (slit width 5 nm). (**B**). Temperature induced changes of light scattering measured at 340 nm of the wild type HspB1 (1) and its R27A (2) mutant.

Under the expression and purification conditions employed, all recombinant proteins were solubly expressed and the combination of two chromatographic steps resulted in a highly purified preparation of both wild type proteins and their point mutants with replacement of the conserved Arg by Ala (Figure 2). In order to analyze the effect of the Arg/Ala substitution we investigated the spectral properties and thermal stability of the wild type proteins and their point mutants. In the case of HspB1, replacement of Arg27 by Ala had no effect on the absorbance and fluorescence spectrum (Figure 3A) or on the temperature of half-maximal unfolding measured by Trp fluorescence and detected at 68 °C for both the wild type protein and its point mutant. However, the amplitude of temperature-induced aggregation of the wild type HspB1 was slightly larger than in the case of its R27A mutant (Figure 3B). Similarly, mutations R21A of HspB4, R22A of HspB5, or R27A of HspB6 had no significant effect either on absorbance in near UV range (270–320 nm) or fluorescence spectra or on temperature-induced aggregation of the analyzed proteins (Supplementary Material Figures S1 and S2). 

**Figure 4:**
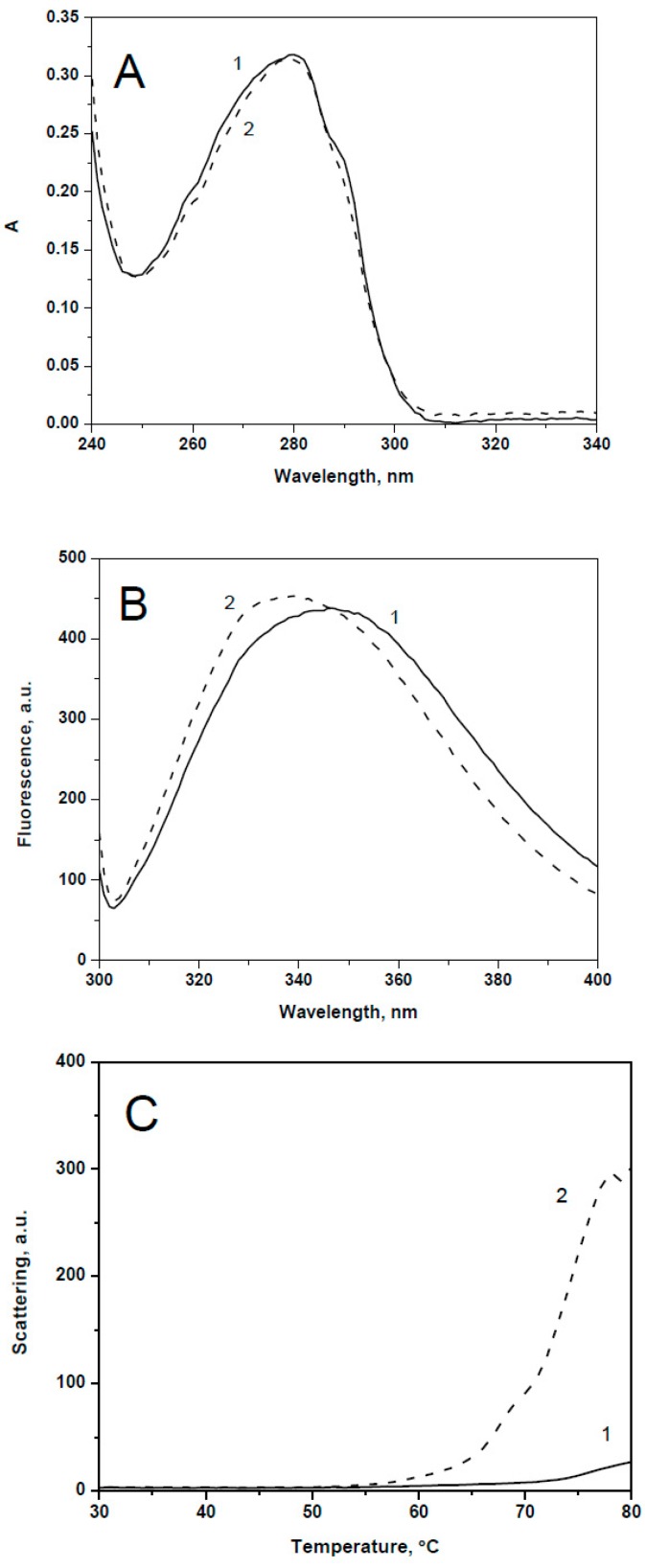
(**A**) Absorbance spectrum of the wild type HspB8 (1) and its R29A (2) mutant. (**B**) Fluorescence spectra of the wild type HspB8 (1) and its Ala mutant (2) excited at 295 nm (slit with 5 nm) and recorded in the range of 300-400 nm (slit width 2.5 nm). (**C**) Temperature induced changes of the light scattering measured at 340 nm of the wild type HspB8 (1) and its R29A (2) mutant.

Mutation R29A of HspB8 was accompanied by the largest changes in spectral properties of this protein. The absorbance spectrum of the wild type HspB8 is characterized by a shoulder at 290–295 nm and this shoulder disappeared in the spectrum of the mutant construct (Figure 4A). Correlated with this, the fluorescence maxima of the wild type HspB8 of 346 nm was blue-shifted to 339 nm in the case of R29A mutant indicating a more hydrophobic environment of Trp residues (Figure 4B). Temperature-induced aggregation of the R29A mutant started at a temperature significantly lower and was much more pronounced than that of the wild type HspB8 (Figure 4C).

Summing up, we might conclude that replacement of the conserved Arg induces spectral changes and/or changes of temperature-induced aggregation only in the case of HspB1 and HspB8, i.e., proteins containing the so-called P1 peptide at the end of the N-terminal domain (Figure 1A). The Arg/Ala substitution had no significant effect on the optical properties and/or temperature-induced aggregation of HspB4, HspB5 and HspB6, i.e., sHsp lacking this peptide.

### 2.2. Effect of Arg/Ala Replacement on sHsp Quaternary Structure

**Figure 5:**
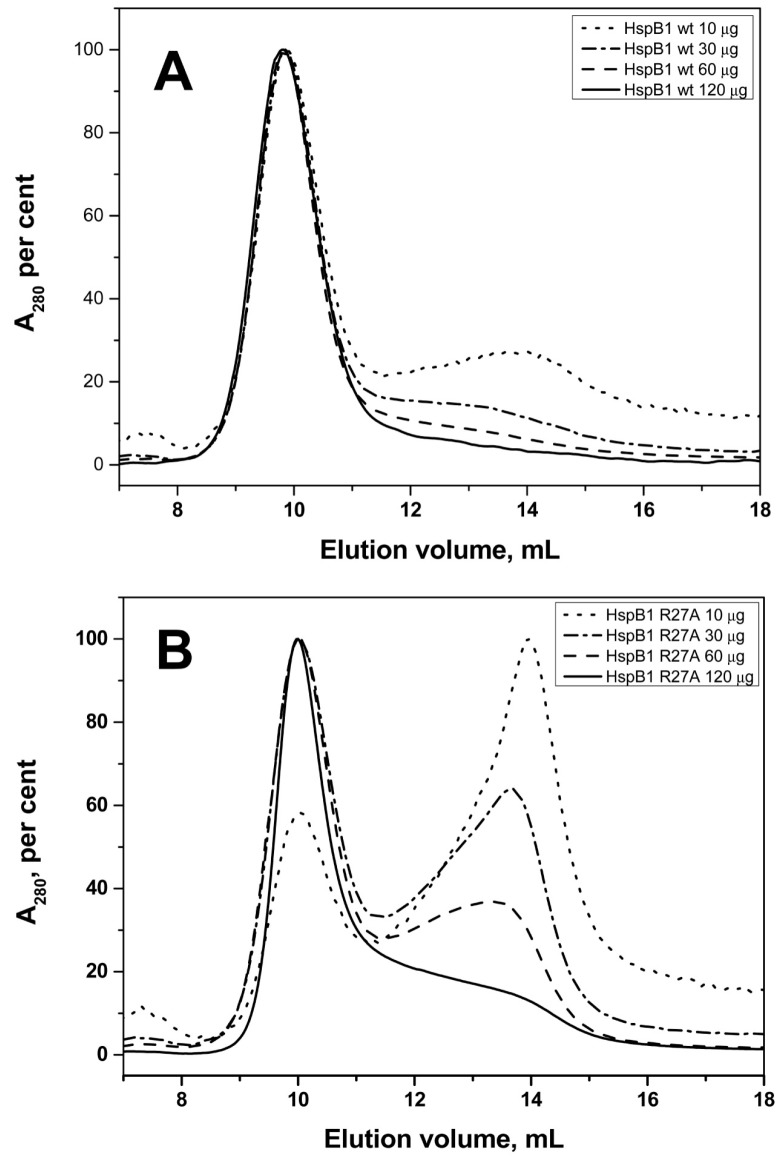

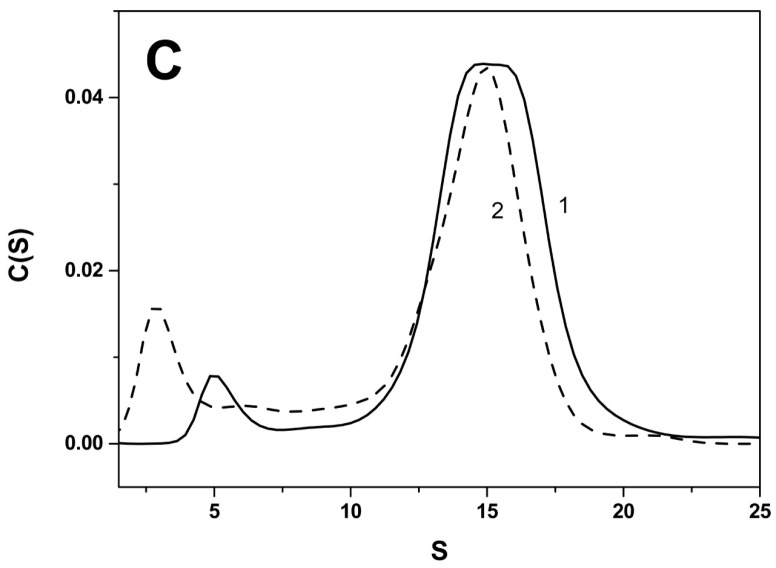
Effect of R27A mutation on quaternary structure of HspB1. Size-exclusion chromatography of the wild type HspB1 (**A**) and its R27A mutant (**B**) Normalized elution profiles obtained after loading on the column 10 (dotted), 30 (dash-dotted), 60 (dashed) and 120 (solid) µg dissolved in 100 µL of buffer are presented. (**C**) Sedimentation velocity analysis of the wild type HspB1 (1) and its R27A mutant (2).

Size-exclusion chromatography (SEC), analytical ultracentrifugation (AUC), and dynamic light scattering (DLS) were used to investigate the quaternary structure of the different sHsp constructs. According to SEC, the wild type HspB1 formed stable high molecular weight oligomers with an apparent molecular weight of ~540 kDa independent of the quantity of protein loaded on the column (Figure 5A, Table 1). The oligomeric structure of the R27A mutant of HspB1 was dependent on the quantity of protein loaded on the column. At low concentrations, the R27A mutant formed two types of oligomer with apparent molecular weight of 80–90 and ~500 kDa, whereas at high concentrations this protein formed only the larger species (Figure 5B). The data of AUC indicate that both wild type HspB1 and its R27A mutant formed two types of oligomers with sedimentation coefficients in the range of 2–7 and 13–17 S (Figure 5C). The ratio between oligomers with high and low sedimentation coefficients was larger in the case of the wild type protein than in the case of the R27A mutant. The average hydrodynamic diameter of the wild type HspB1 (11.5 ± 0.5 nm, number of measurements, *n* = 110) was slightly larger, than the corresponding diameter (11.1 ± 0.5 nm, *n* = 130) of R27A mutant. A change that was statistically significant (*t* test value equal to 5).

Size-exclusion chromatography (SEC), analytical ultracentrifugation (AUC), and dynamic light scattering (DLS) were used to investigate the quaternary structure of the different sHsp constructs. According to SEC, the wild type HspB1 formed stable high molecular weight oligomers with an apparent molecular weight of ~540 kDa independent of the quantity of protein loaded on the column (Figure 5A, Table 1). The oligomeric structure of the R27A mutant of HspB1 was dependent on the quantity of protein loaded on the column. At low concentrations, the R27A mutant formed two types of oligomer with apparent molecular weight of 80–90 and ~500 kDa, whereas at high concentrations this protein formed only the larger species (Figure 5B). The data of AUC indicate that both wild type HspB1 and its R27A mutant formed two types of oligomers with sedimentation coefficients in the range of 2–7 and 13–17 S (Figure 5C). The ratio between oligomers with high and low sedimentation coefficients was larger in the case of the wild type protein than in the case of the R27A mutant. The average hydrodynamic diameter of the wild type HspB1 (11.5 ± 0.5 nm, number of measurements, *n* = 110) was slightly larger, than the corresponding diameter (11.1 ± 0.5 nm, *n* = 130) of R27A mutant. A change that was statistically significant (*t* test value equal to 5).

The wild type HspB4 and its R21A mutant both formed stable large size oligomers with an apparent molecular weight of ~590 kDa. The oligomeric size did not vary when changing the concentration of protein loaded on the column (Supplementary Material Figure S3). The data of AUC indicate that HspB4 forms a heterogeneous mixture of small 2–3 S and large 12–20 S oligomers. According to AUC, both the wild type HspB4 and its R21A mutant presented a mixture of large oligomers with sedimentation coefficients of 13, 16, and 19 S (Supplementary Material Figure S3). The small difference in oligomer distribution was confirmed by DLS data indicating that the hydrodynamic radius of the wild type HspB4 was equal to 12.1 ± 0.6 nm (*n* = 130), whereas the corresponding value for R21A mutant was equal to 11.8 ± 0.9 nm (*n* = 130).

**Figure 6:**
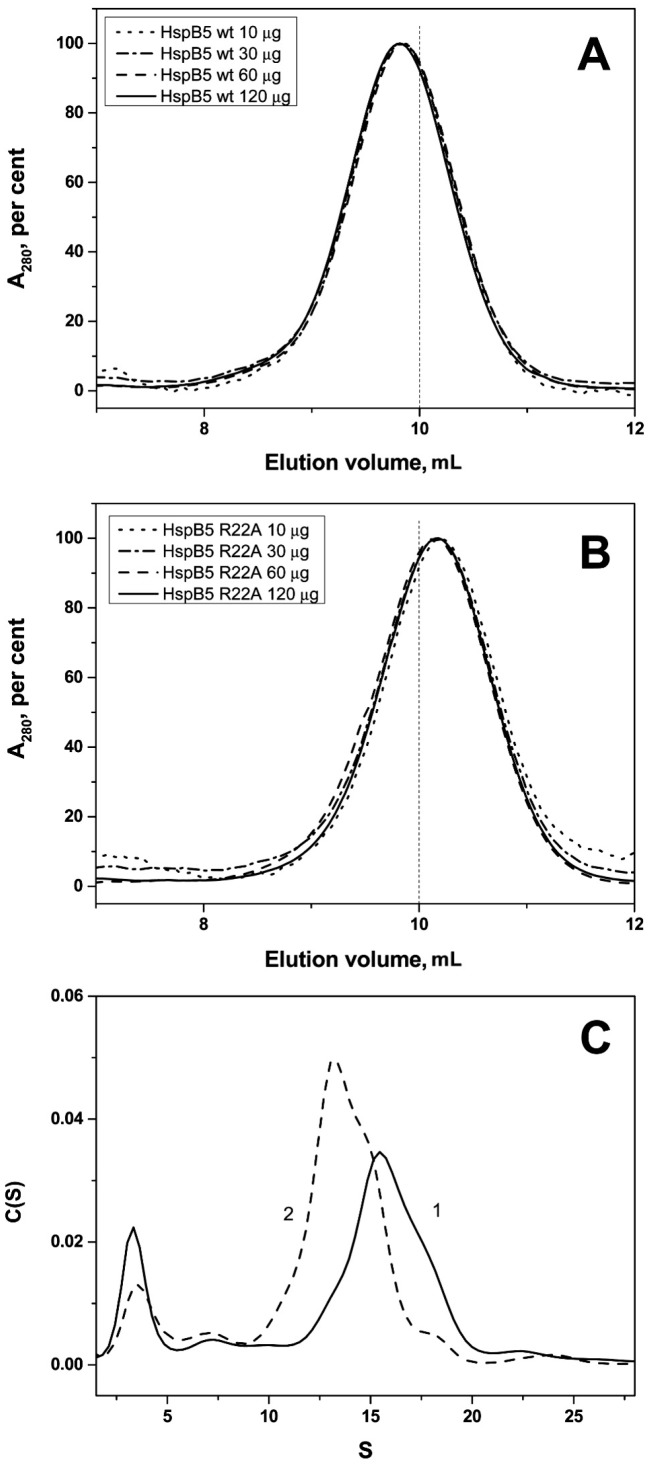
Effect of R22A mutation on quaternary structure of HspB5. Size-exclusion chromatography of the wild type HspB5 (**A**) and its R22A mutant (**B**) Normalized elution profiles obtained after loading on the column 10 (dotted), 30 (dash-dotted), 60 (dashed) and 120 (solid) µg dissolved in 100 µL of buffer are presented. (**C**) Sedimentation velocity analysis of the wild type HspB5 (1) and its R22A mutant (2).

The SEC profiles indicate that the wild type HspB5 formed large stable oligomers with apparent molecular weight of ~540 kDa similar to those formed by the wild type HspB1 and slightly smaller than those formed by HspB4 (Figure 6). The R22A mutant of HspB5 also formed stable oligomers with a slightly lower apparent molecular weight of ~460 kDa. In both cases, the size of oligomers was independent of protein concentration loaded on the column (Figure 6). Two peaks were detected on the sedimentogram of the wild type HspB5 and its point mutant. The first small peak with sedimentation coefficient of 3.0–3.5 S probably corresponds to small oligomers (probably crystalline tetramers). The second peak with sedimentation coefficient ~16 S for the wild type HspB5 and sedimentation coefficient ~14 S for R22A mutant of HspB5 correspond to the large oligomers of these proteins (Figure 6). The hydrodynamic diameter determined by DLS was equal to 11.8 ± 0.6 nm (*n* = 150) for the wild type protein and 11.1 ± 0.4 nm (*n* = 130) for its R22A mutant.

Human wild type HspB6 formed only small size oligomers with an apparent molecular weight of ~50 kDa on SEC (Supplementary Material Figure S4). An increase in the concentration of loaded sample was accompanied by minimal changes of the apparent molecular weight. Similar results were obtained with the R27A mutant of HspB6 (Supplementary Material Figure S4). Both the wild type HspB6 and its R27A mutant had a similar sedimentation coefficient ~2.5–3.0 S, and their hydrodynamic diameter was equal 5.9 ± 0.8 nm (*n* = 130). 

**Figure 7:**
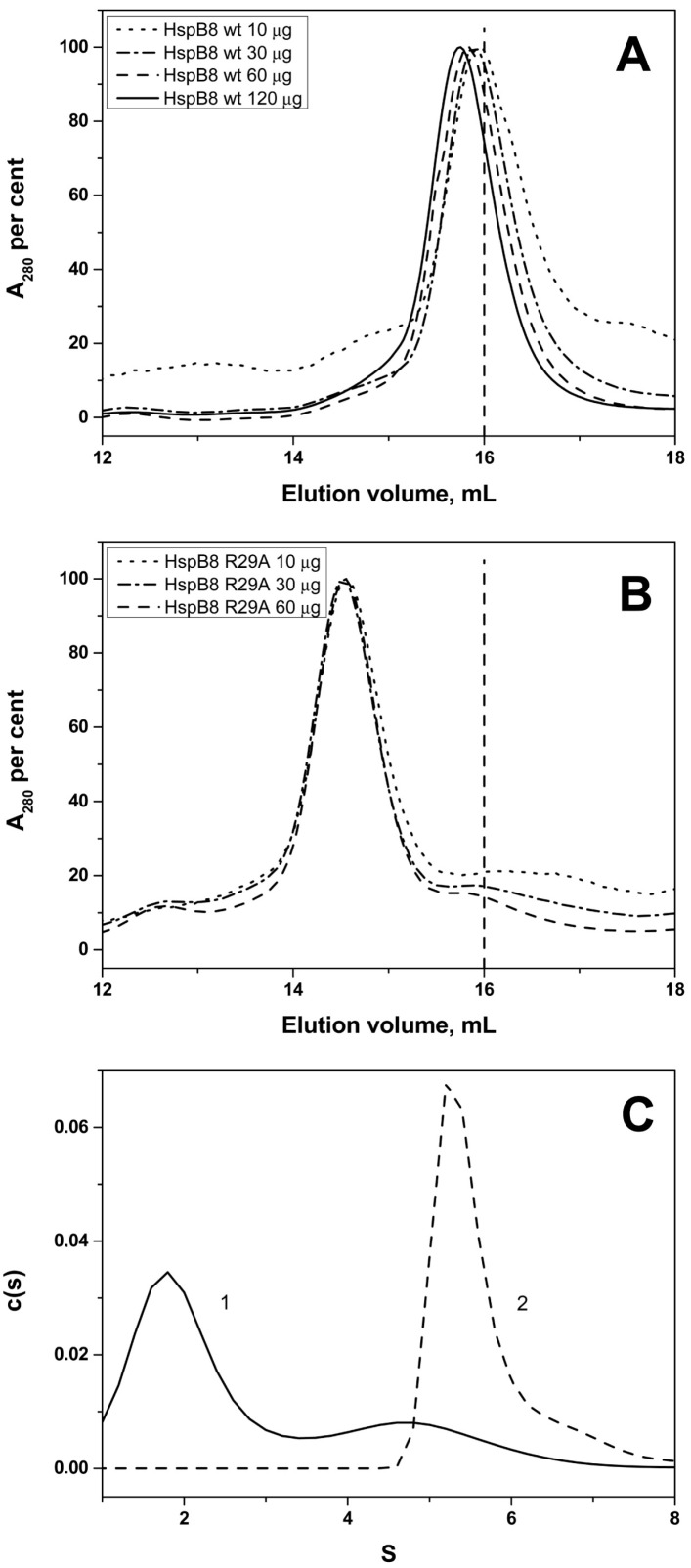
Effect of R29A mutation on quaternary structure of HspB8. Size-exclusion chromatography of the wild type HspB8 (**A**) and its R29A mutant (**B**) Normalized elution profiles obtained after loading on the column 10 (dotted), 30 (dash-dotted), 60 (dashed) and 120 (solid) µg dissolved in 100 µL of buffer are presented. (**C**) Sedimentation velocity analysis of the wild type HspB8 (1) and its R29A mutant (2).

Like HSPB6, the wild type HspB8 also formed small oligomers with an apparent molecular weight of 33 kDa on SEC (Figure 7). The elution volume of wild type HspB8 was only weakly dependent on the quantity of protein loaded on the column. At the same time the R29A mutant of HspB8 eluted with an apparent molecular weight of ~60 kDa and its elution volume was again independent of the quantity of protein loaded on the column (Figure 7). The smallest oligomer formed by the wild type HSPB8 was previously suggested to be a dimer [35]. Since the obtained SEC-based apparent mass value was significantly deviating from that of the expected dimer, we additionally performed an online SEC-SAXS experiment using synchrotron radiation (Supplementary Material Figure S5). When 230 µM of sample was applied, the separation on the SEC column resulted in one major peak. The resulting SAXS curve, and corresponding dimensionless-Kratky and pair-distribution plots suggest that the protein contains a core folded region with extended disordered regions similar to what was previously reported for HspB6 [33]. Mass estimates at the elution peak center resulted in a value of 49 kDa using the volume of correlation invariant [36]. Hence, we conclude that the main species of the wild type HspB8 in a moderately concentrated solution is a dimer, whereas the mutation causes an approximate doubling of the mass, i.e., a tetramer. These unexpected results were confirmed by AUC where the sedimentation coefficient of the wild type HspB8 was determined to be equal to 2.5 S, whereas the R29A mutant was sedimented with a coefficient of 5.8 S (Figure 7). These data also agree with results obtained by DLS indicating that the hydrodynamic diameter of the wild type HspB8 is equal to 5.6 ± 0.4 nm (*n* = 130), whereas the hydrodynamic diameter of its R29A mutant is equal to 6.2 ± 0.4 nm (*n* = 130). 

**Figure 8:**
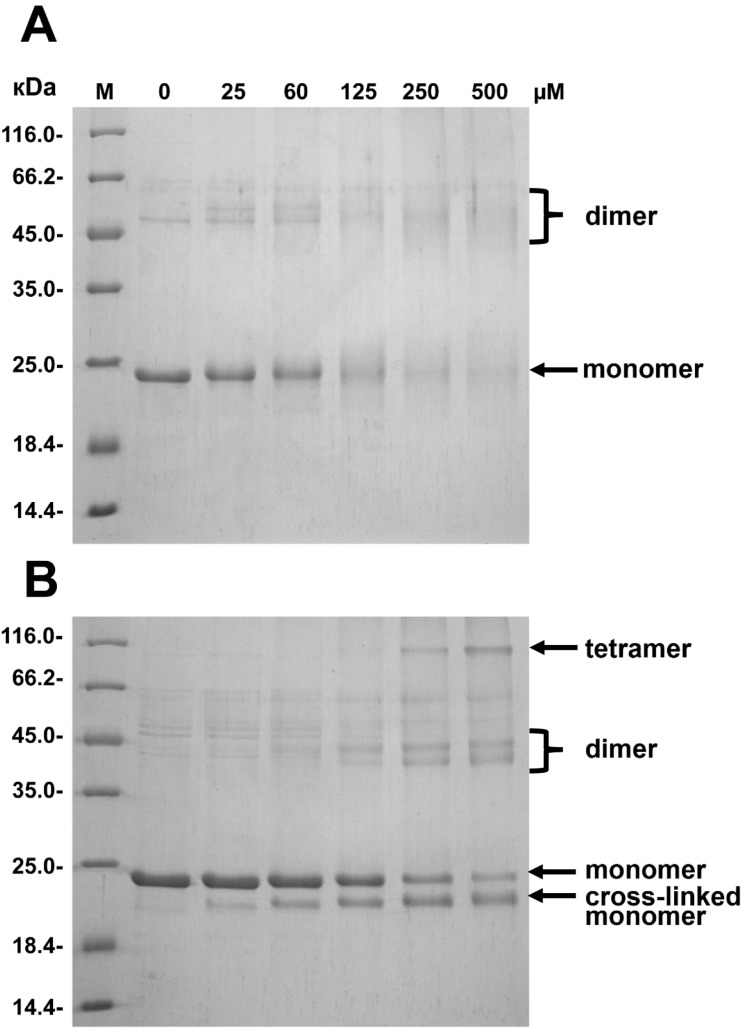
Chemical crosslinking of the wild type HspB8 (**A**) and its R29A mutant (**B**) by DSS. The wild type HspB8 and its R29A mutant (1.3 mg/mL) were incubated for 30 min in PBS with different concentrations of DSS (indicated above each track). Molecular weight markers were loaded on track labeled M.

The changes in the quaternary structure of HspB8 upon mutation could be further confirmed using chemical cross-linking. Incubation with DSS was accompanied by accumulation of both intramonomer and intermonomer cross-linked proteins (Figure 8). Cross-linking of the wild type protein resulted in accumulation of very diffuse bands corresponding to cross-linked monomers (apparent molecular weight 22 kDa) and dimers (apparent molecular weight ~45 kDa) (Figure 8A). At the same time cross-linking of the R29A mutant resulted in accumulation of cross-linked monomer (apparent molecular weight 22 kDa) as well as cross-linked dimers (apparent molecular weight ~45 kDa) and cross-linked oligomers with high apparent molecular weight (about 116 kDa) that were absent in the case of the wild type protein (Figure 8B). Qualitatively similar results were obtained when HspB8 and its mutant were subjected to mild oxidation of SH groups (data not presented). 

### 2.3. Lack of the Effect of Arg/Ala Replacement on Chaperone-Like Activity

**Figure 9:**
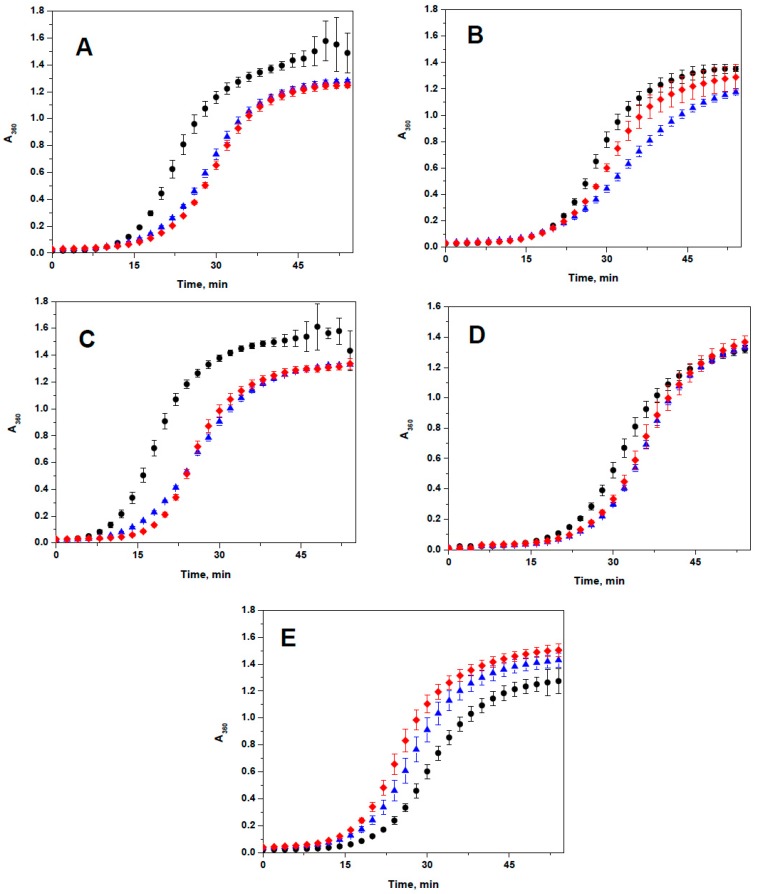
Chaperone-like activity of HspB1 (**A**), HspB4 (**B**), HspB5 (**C**), HspB6 (**D**) and HspB8 (**E**) with ovotransferrin as a model substrate. Kinetics of aggregation of isolated ovotransferrin (black circles) and ovotransferrin in the presence of the wild type protein (blue triangles) or aggregation of ovotransferrin in the presence of corresponding R/A mutant (red diamonds) were recorded in no less than three independent experiments and data presented are mean with error bars corresponding to standard deviation. Weight ratio ovotransferrin/sHsp was equal to 50 for HspB1, 11 for HspB4, 17 for HspB5, 14 HspB6, and 15 for HspB8.

Heating of apo-ovotransferrin leads to its denaturation and aggregation (Figure 9). HspB1, HspB4, HspB5, and HspB6 inhibited ovotransferrin aggregation and chaperone-like activity of the wild type proteins was comparable with that of the corresponding R to A mutants (Figure 9). Only in the case of HspB4 was the wild type protein slightly more effective than its corresponding R21A mutant. At the same time, HspB8 and its R29A mutant were unable to inhibit ovotransferrin aggregation and instead slightly increased aggregation of this model substrate (Figure 9E). 

Similar results were obtained when another model substrate, myosin subfragment-1, was used for estimation of chaperone-like activity. Again HspB1, HspB4, HspB5, and HspB6 demonstrated chaperone-like activity and this activity was comparable for both the wild type protein and its Arg/Ala mutants. HspB8 and its point mutant did not inhibit heat-induced aggregation of S-1 fragment of myosin and even slightly increased its aggregation demonstrating anti-chaperone activity (Supplementary Material Figure S6).

## 3. Discussion

Human sHsps are known to have major differences in their ability to form large oligomers. In particular, HspB1, HspB4, and HspB5 form species with an apparent molecular weight of more than 500 kDa, while HspB2 and HspB3 form hetero-tetramers, and HspB6 and HspB8 form smaller entities. The tendency to form large oligomers is known to correlate with the presence of a conserved IXI motif in the CTD [7,8,37]. Of the five proteins studied here, this is only the case in HspB1, HspB4, and HspB5. Although this motif plays an important role in the interaction of sHsp dimers within the large oligomers, stability of these large oligomers appears to be mostly dependent on the structure and properties of their respective NTDs. The detailed location of the NTD within the oligomeric assembly is a subject of a debate, but it is likely that the NTDs are buried in the core of the oligomer. Interacting with each other and probably with the ACDs, the NTDs stabilize the overall structure of these oligomers [16,38,39]. Indeed, point mutations [40] and posttranslational modifications [12,16,41] affect the oligomeric structure of HspB1 and HspB5. Specifically, removal of the overall positive charge in the NTD often leads to the dissociation of large sHsp oligomers, as evident from the data on phosphorylation and phosphomimicking mutations of Ser15, Ser78, and Ser82 in HspB1, and Ser19, Ser 45, and Ser79 in HspB5 [12,16]. On the flip side, introduction of excessive positive charges as a rule prevents or decreases dissociation induced by phosphorylation. For instance, G34R and E41K mutants of HspB1 are much more resistant to phosphorylation-induced dissociation than the wild type protein [29], and replacement of certain negatively charged residues by cysteine in the NTD of a triple serine to aspartate mutant of HspB1 yielded large oligomers [40]. 

We demonstrate here that the replacement R/A in the conserved RLFDQxFG motif of HspB1 and HspB5, i.e., decrease of positive charge in the NTD, provokes destabilization of large oligomers of HspB1 (Figure 5) and decreases the size of large oligomers of HspB5 (Figure 6). HspB4 forms a heterogeneous mixture of different oligomers (Supplementary Material Figure S3), and therefore it was difficult to follow the changes induced by the R21A mutation to its structure. At the same time it is worthwhile mentioning that mutations R21L, R21W, or R21Q in HspB4 are associated with cataract [42,43,44,45,46], suggesting that a mutation of residue R21 could affect the interaction of HspB4 with HspB5. 

Both HspB6 and HspB8 lack the IP(V/I) motif in the CTD and are known to have a much reduced tendency to form large oligomers compared to other human sHsps [33,34,47]. We show that the R27A mutation induced only small changes in the optical properties or oligomeric structure of HspB6 (Supplementary Material Figures S1 and S2). These results agree with the earlier published data indicating that the point mutations Q31A and Q31L have only a moderate effect on the quaternary structure of HspB6 [24] and that phosphorylation (or phosphomimicking mutation) of Ser16 affects the quaternary structure of HspB6 only under crowding conditions [48,49]. Although the R27A mutation described in this work and the larger deletion of residues 21–30 or 31–40 do not significantly affect oligomeric structure of isolated HspB6 [24], the conserved motif RLFDQxFG seems to play crucial role in formation of heterooligomeriс complexes of HspB6 and HspB1 [27].

Through a combination of biophysical methods, including SEC-SAXS experiments, we could demonstrate that at moderate concentrations HspB8 is predominantly present as dimers in solution, which is in line with earlier data [35,50]. Proteomics investigations indicate that this protein can be phosphorylated at Ser24 and Thr/Ser87 by different protein kinases in vivo [17,51]. Phosphorylation or phosphomimicking mutations of Ser24, Ser27, and Thr87 are accompanied by changes of Trp fluorescence, susceptibility to limited proteolysis, and an increase in the apparent molecular weight of HspB8 [52]. This means that in the case of HspB8 introduction of negative charges induces large changes in the structure and/or orientation of the NTD. Here we show that the replacement of positively charged Arg by Ala results in a significant increase in the apparent molecular weight and sedimentation coefficient (Figure 7). Cross-linking experiments indicate that R29A mutation was accompanied by the accumulation of large oligomers with apparent molecular weight of more than 116 kDa that were not observed for the wild type protein (Figure 8). It seems likely that this mutation in HspB8 stabilizes the structure of the intrinsically disordered NTD, which promotes the association of dimers into tetrameric species.

Finally, analysis of chaperone-like activity has not revealed dramatic changes induced by replacement of conserved Arg (Figure 9). However, depending on substrate nature, all five sHsp demonstrated different chaperone (or antichaperone in the case of HspB8) activity. In this respect our results correlate with the recently published data of Mymrikov et al. [53].

Phylogenetic analysis indicates that the five sHsps belong to two different clades (Figure 1B) [18,54]. HspB1 and HspB8 are closely related and belong to one clade, whereas HspB4, HspB5, and HspB6 belong to another clade. The main difference between these two clades is a rather large insert at the end of the N-terminal domain (residues 59–73 of HspB1 and residues 62–75 of HspB8) which is only present in the first clade. This insert (marked as P1) is poorly conserved and rich in Pro. This part of the NTD was hypothesized to play a role in the formation of large HspB1 oligomers [55]. Of note, the replacement of Arg by Ala in the conserved RLFDQxFG motif was accompanied by pronounced changes in spectral properties and/or temperature-induced aggregation only in the sHsp belonging to the first clade (HspB1 and HspB8). In HspB1, this replacement has apparently lead to some destabilization of the HspB1 oligomers (Figure 5). However, the mutation had an opposite effect in normally dimeric HspB8, inducing the formation of tetramers. It seems surprising that the single point mutation induces significant changes in the oligomeric state of HspB8. In this respect, it is worthwhile to mention that highly homologous human and rat HspB6 containing only a few substitutions in the NTD differ in their ability to form oligomers. Indeed, rat HspB6 tends to form concentration-dependent oligomers [56], whereas human HspB6 is predominantly presented by dimers [33,49]. Thus our data indicate that the effect of the conserved RLFDQxFG motif on the oligomeric structure depends on the context of the particular sHsp. 

## 4. Materials and Methods

### 4.1. Proteins

A pET23b construct containing the human HspB1, HspB4, HspB5, HspB6, and HspB8 sequences were used by Eurogen (Moscow, Russia) for generating expression constructs encoding R27A HspB1, R21A HspB4, R22A HspB5, R27A HspB6, and R29A HspB8 mutants. The integrity of each construct was confirmed by sequencing. All proteins were expressed in the BL21(DE3)pLysS *E. coli* strain by culturing either in LB at 37 °C and inducing production with 0.5 mM IPTG (HspB1, HspB4, HspB5, HspB8) or by using auto-induction media at the same temperature (HspB6) [57]. Earlier described methods were used for purification of the wild type proteins and their mutants [49,58]. Briefly, the bacterial lysate was subjected to ammonium sulfate fractionation, followed by ion-exchange chromatography on HiTrap Q HP (in the case of HspB1, HspB4, and HspB5) or hydrophobic chromatography on HiTrap Phenyl Sepharose (in the case of HspB6 and HspB8). Finally all proteins were subjected to size-exclusion chromatography (SEC) using a Superdex-200 (GE Healthcare, Uppsala, Sweden). According to SDS-PAGE analysis [59], the purity of all protein samples was higher than 95%. All proteins were aliquoted and stored at −20 °C in buffer B (20 mM Tris/acetate pH 7.6 containing 10 mM NaCl, 0.1 mM EDTA, 0.1 mM PMSF and 2 mM DTT).

### 4.2. Fluorescence Spectroscopy and Light Scattering

Fluorescence of all proteins (0.05–0.15 mg/mL) was measured at 25 °C in buffer F containing 20 mM HEPES/NaOH pH 7.6, 100 mM NaCl, 0.5 mM EDTA, and 2 mM DTT. Fluorescence was excited at 295 nm (slit width 5 nm) and recorded in the range of 310–400 nm (slit width 5 nm). 

Heat-induced aggregation was monitored by heating of protein samples (0.3 mg/mL) in buffer F with a constant rate of 1 °C/min from 25 to 90 °C. The samples were illuminated at 340 nm (slit width 2.5 nm) and signal was recorded at 340 nm (slit with 2.5 nm). All fluorescent and static light scattering experiments were performed on a Varian Carry Eclipse spectrofluorometer (Agilent, Santa Clara, CA, USA).

Dynamic light scattering was performed on Zetasizer Nano ZS (Malvern Panalytical, Malvern, UK). All protein solutions were prepared in buffer F filtered through 0.2 µm filter. Experiments were performed at 25 °C at protein concentration equal to 0.3 mg/mL for HspB1, HspB5, and HspB4 and at concentration equal to 0.6 mg/mL for HspB6 and HspB8. Each experiment lasted for 10 s and was repeated 15 times. This cycle was repeated 10 times, thus providing the accumulation of 150 measurements. Number distribution was used for calculation of hydrodynamic diameter with Zetasizer software.

### 4.3. Size-Exclusion Chromatography

The quaternary structure was analyzed by means of size-exclusion chromatography (SEC) performed on a Superdex 200 HR 10/30 column equilibrated with buffer containing 20 mM Tris/acetate (pH 7.6), 150 mM NaCl, 0.1 mM EDTA, 0.1 mM PMSF, and 15 mM β-mercaptoethanol. Samples containing 10–120 µg of protein dissolved in 100 µL of the same buffer were loaded on the column and eluted with the rate 0.5 mL/min. The column was calibrated with thyroglobulin (660 kDa), ferritin (440 kDa), aldolase (158 kDa), conalbumin (75 kDa), ovalbumin (43 kDa), carboanhydrase (29 kDa), and RNAse (13.7 kDa).

### 4.4. Analytical Ultracentrifugation

The wild type proteins and their point mutants at equal concentration (0.4 mg/mL for HspB1 and HspB8 and 0.8 mg/mL for HspB4, HspB5, and HspB6) were diluted and dialyzed against 50 mM phosphate buffer (pH 6.8), containing 150 mM NaCl and 2 mM DTT. The samples were subjected to ultracentrufugation (42,000 rpm) in a Spinco E analytical ultracentrifuge at 20 °C in a six—hole AnJ-Ti rotor and 12 mm double sector cells. All cells were scanned at 280 nm with a 3 min interval between measurements. Sedimentation coefficients were estimated from differential distribution of sedimentation coefficients [c(s,f/f_o_) vs. s] using SEDFIT program [60].

### 4.5. SEC-SAXS Studies

SAXS experiments were performed on the SWING beamline at the Synchrotron Soleil [61] essentially as described before [24]. Briefly, a 75 mL sample of wild-type HspB8 at 5 mg/mL was loaded onto a Shodex KW404-4F pre-equilibrated in 20 mM HEPES, 150 mM NaCl, pH 7.4, and 2.5 mM DTT. Scattering data of the column eluent was collected using a 0.75 s exposure, with a 1 s delay between frames. Initial data reduction was performed using FOXTROT (available at the SWING beamline, https://www.synchrotron-soleil.fr/en/beamlines/swing) and processed further using ScÅtter (available online: http://www.bioisis.net/tutorial), the output of the processed data is presented in Supplementary Material Figure S5.

### 4.6. Crosslinking of HspB8

Two approaches were used for cross-linking of HspB8 and its mutant. Firstly, disulfide bridge formation was examined. The sample was subjected to centrifugation (12,000× *g*, 15 min). The concentrations of the wild type proteins and its mutant were equalized (1.2 mg/mL) and both samples were dialyzed against buffer B (20 mM Tris/acetate pH 7.6, 10 mm NaCl, 0.1 mM EDTA, and 0.1 mM PMSF) at 37 °C for different amounts of time lasting from 2 up to 24 h. HspB8 contains 3 Cys residues and mild oxidation leads to formation of both cross-linked monomers and cross-linked oligomers [35]. The reaction was stopped by the addition of an excess of iodoacetamide. The oligomeric state of the samples thus obtained was analyzed by SDS-gel electrophoresis [59].

Secondly, HspB8 and its mutant were cross-linked by suberic acid bis(*N*-hydroxysuccinimide ester) (DSS). The samples of wild type HspB8 and its mutant were reduced by addition of 5 mM DTT and incubated for 10 min at 37 °C. The protein samples were desalted and transferred to PBS by using Zeba Micro Spin Desalting Columns. The protein concentration was equalized (1.6 mg/mL) and both proteins were incubated in PBS for 30 min at 37 °C with DSS concentration equal to 0, 25, 60, 125, 250, and 500 µM. The reaction was stopped by the addition of Tris, up to the final concentration of 20 mM, and afterwards subjected to SDS-PAGE analysis [59].

### 4.7. Chaperone-Like Activity

Two model protein substrates, chicken apo-ovotransferrin and subfragment-1 of rabbit skeletal muscle myosin, were used for determination of chaperone-like activity. Chicken ovotransferrin was purified as described earlier [62] and its apoform was prepared according to Evans and Holbrook [63]. All experiments were performed in 50 mM phosphate pH 7.4, containing 100 mM NaCl and 20 mM DTT at 45 °C. Small heat shock proteins (final concentration 0.010–0.045 mg/mL) were preincubated in reaction mixture for 8 min and the reaction was started by addition of apo-ovotransferrin (final concentration 0.5 mg/mL). Kinetics of ovotransferrin aggregation was followed by measuring optical density at 360 nm on an Ultrospec 3100 spectrophotometer (GE Healthcare, Uppsala, Sweden). All experiments were repeated no less than three times and results presented are mean ± SD.

Subfragment-1 of rabbit skeletal myosin was obtained as described earlier [64] and was kindly provided by Professor D.I. Levitsky (Institute of Biochemistry, Russian Academy of Sciences). All experiments were performed in 20 mM HEPES/NaOH pH 7.0, 115 mM KCl, and 20 mM DTT at 42 °C. Small heat shock proteins were incubated in the abovementioned buffer for 8 min in order to reduce all sulfhydryl groups and the reaction was started by addition of subfragment-1 (S1) up to the final concentration of 0.4 mg/mL. Aggregation of S1 was followed by measuring optical density at 340 nm in the absence or in the presence (0.02–0.04 mg/mL) of different small heat shock proteins. All experiments were repeated no less than three times and results presented are mean ± SD.

## Figures and Tables

**Table 1:** Apparent molecular weight and estimated number of subunits in oligomers of different small heat shock proteins determined by size-exclusion chromatography (SEC) and SEC-SAXS.

Protein	Monomeric Mass, kDa	Apparent Mass/No. of Monomers WT	Apparent Mass/No. of Monomers Mutant
HspB1	22.8	540/~24	500/~22 + 80/~4 *
HspB4	19.9	590/~30	590/~30
HspB5	20.2	540/~27	460/~23
HspB6	17.1	50/~3 **	50/~3
HspB8	21.6	33/~2 ***	60/~3

* smaller species at low concentration; ** predominantly a dimer [33,34]; *** predominantly a dimer according to SEC-SAXS measurements (this work).

## Supplementary Materials

Supplementary materials can be found at http://www.mdpi.com/1422-0067/19/7/2112/s1.
