# Supplementary material for: The Role of the Arginine in the Conserved N-Terminal Domain RLFDQxFG Motif of Human Small Heat Shock Proteins HspB1, HspB4, HspB5, HspB6, and HspB8
Source: Int J Mol Sci. 2018 Jul 20;19(7):2112. doi: 10.3390/ijms19072112 (PMC6073470; doi:10.3390/ijms19072112)
Supplement: Supplementary file 1 [file ijms-19-02112-s001.pdf]

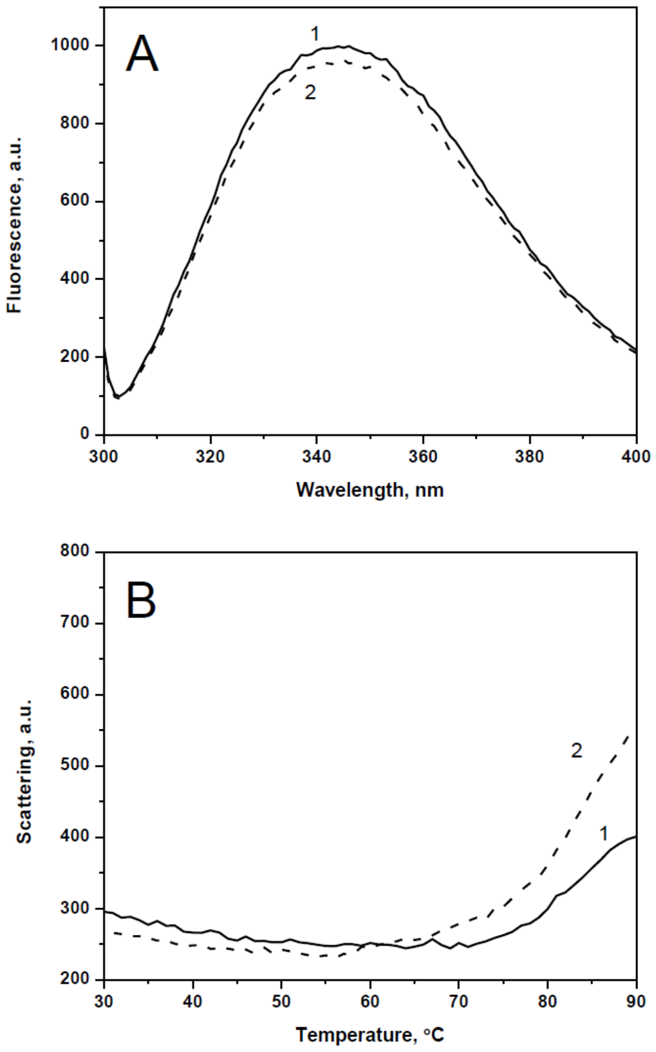

Supplementary Material Figure S1. (A). Fluorescence spectra of the wild type HspB6 (1) and its R27A (2) mutant excited at 295 nm (slit width 5 nm) and recorded in the range of 300-400 nm (slit width 5 nm). **(B).** Temperature induced changes of light scattering measured at 340 nm of the wild type HspB6 (1) and its R27A (2) mutant.

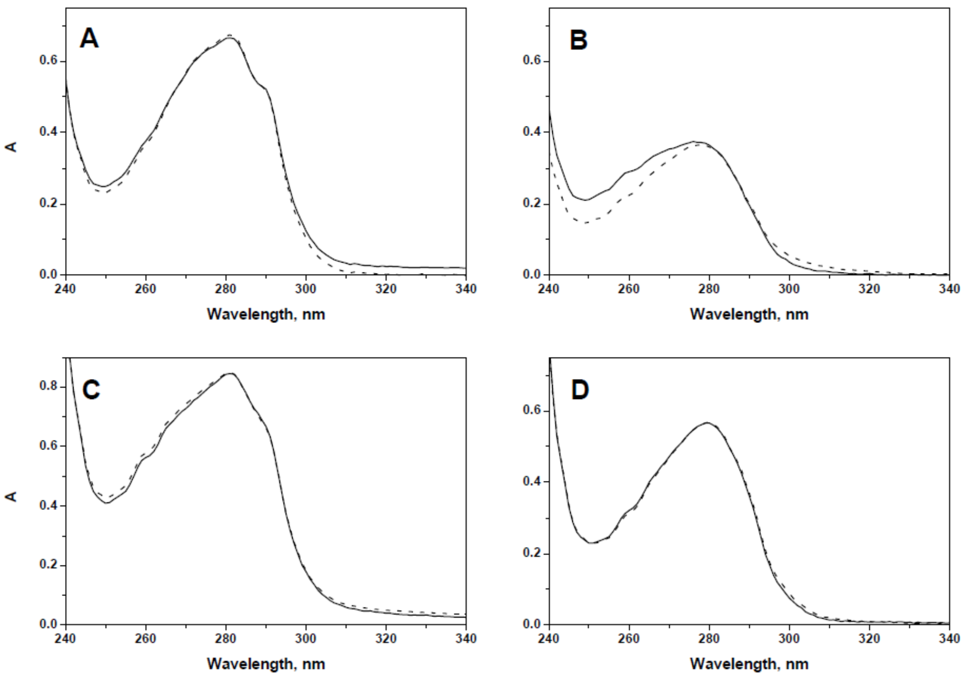

Supplementary Figure S2. UV-absorbance spectra of HspB1 (A), HspB4 (B), HspB5 (C) and HspB6 (D). Solid and dashed curves correspond to spectra of the wild type and mutated proteins respectively.

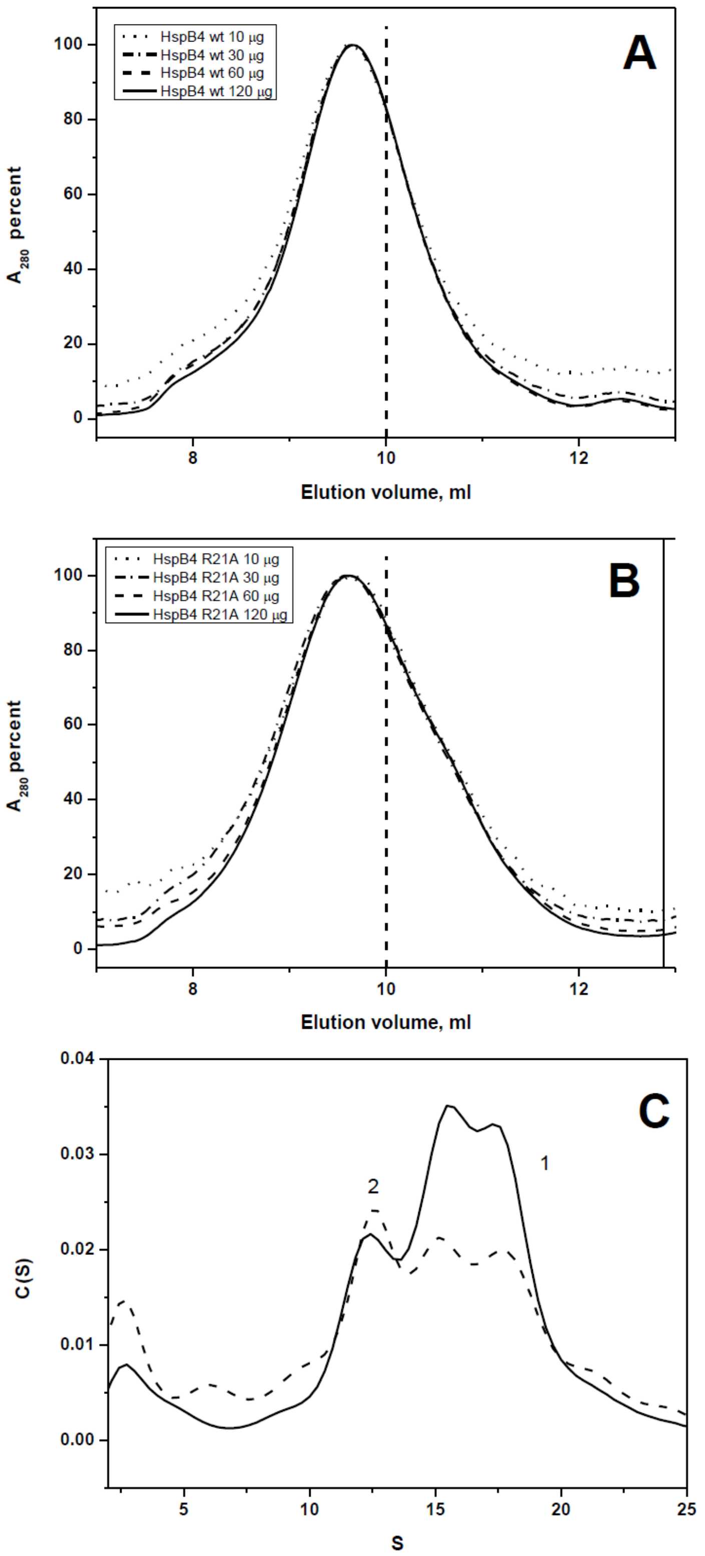

Supplementary Material Figure S3. Effect of R21A mutation on quaternary structure of HspB4. A and B. Size-exclusion chromatography of the wild type HspB4 **(A)** and its R21A **(B)**. Normalized elution profiles obtained after loading on the column 10 (dotted), 30 (dash-dotted), 60 (dashes) and 120 (solid) µg dissolved in 100 µl of buffer are presented. (**C**). Sedimentation velocity analysis of the wild type HspB4 (1) and its R21A mutant (2).

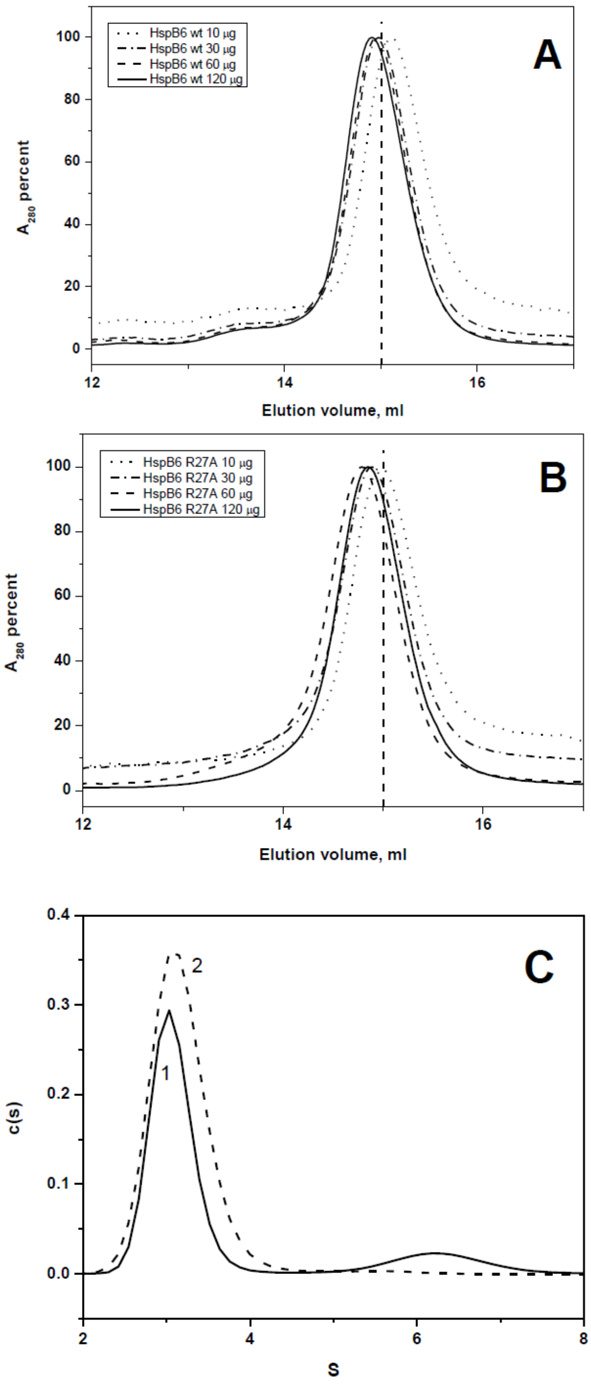

Supplementary Material Figure S4. Effect of R27A mutation on quaternary structure of HspB6. A and B. Size-exclusion chromatography of the wild type HspB6 **(A)** and its R27A mutant **(B).** Normalized elution profiles obtained after loading on the column 10 (dotted), 30 (dash-dotted), 60 (dashes) and 120 (solid) µg dissolved in 100 µl of buffer are presented. (**C).** Sedimentation velocity analysis of the wild type HspB6 (1) and its R27A mutant (2).

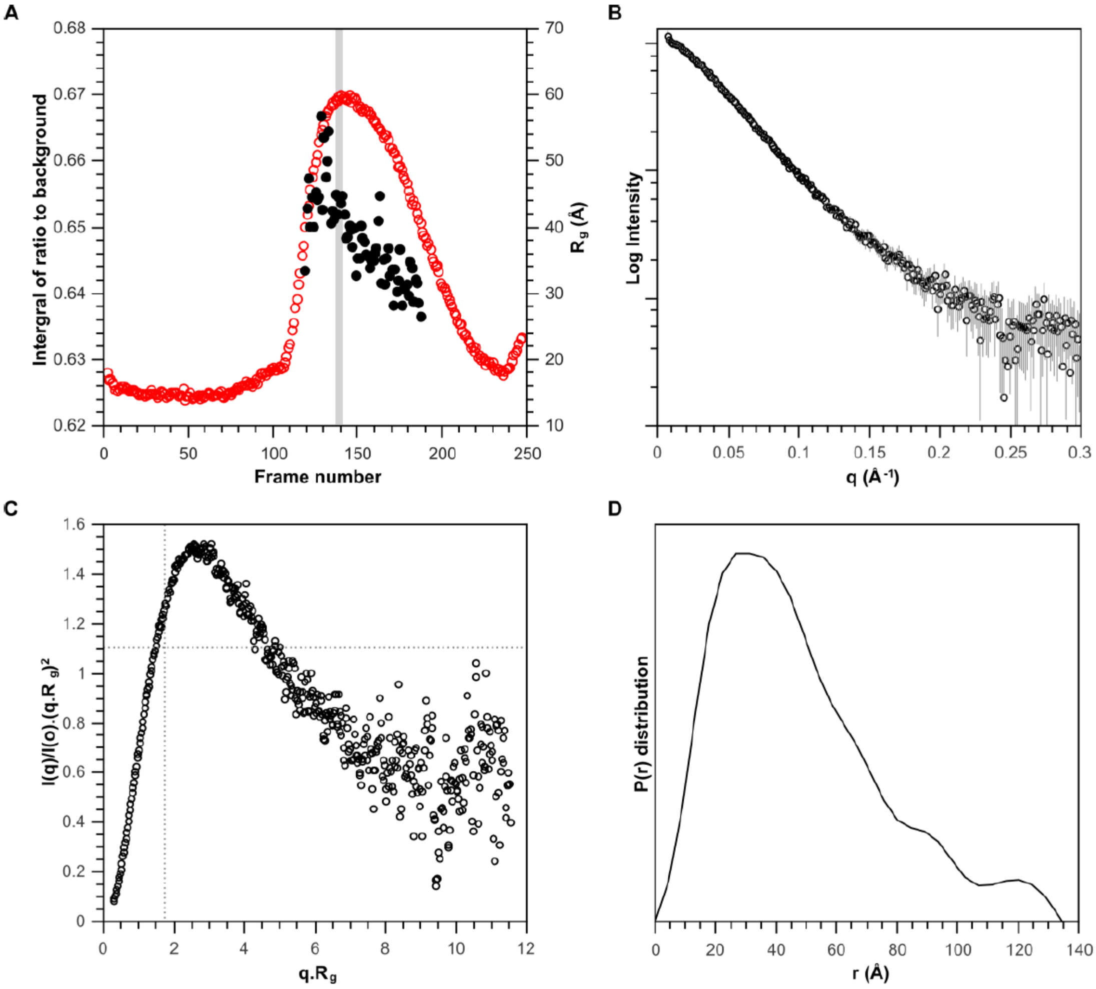

Supplementary Materials Figure 5. SAXS analysis of HSPB8 (A) SEC-SAXS Signal Plot. Each point (red circles) represents the integrated area of the ratio of the specific frame SAXS curve to the estimated background. The calculated radius of gyration (R_g) for each SAXS curve across the eluting protein peak is shown (black filled circles). The grey shaded box corresponds to frames scaled and averaged together to generate the final SAXS curve. **(B)** Averaged SAXS curve of HSPB8. (**C)** Dimensionless Kratky plot. The cross-hair marks the Guinier-Kratky point (1.732, 1.1) corresponding to the peak position of a globular particle (**D)** Pair-distance, P(r), distribution function.

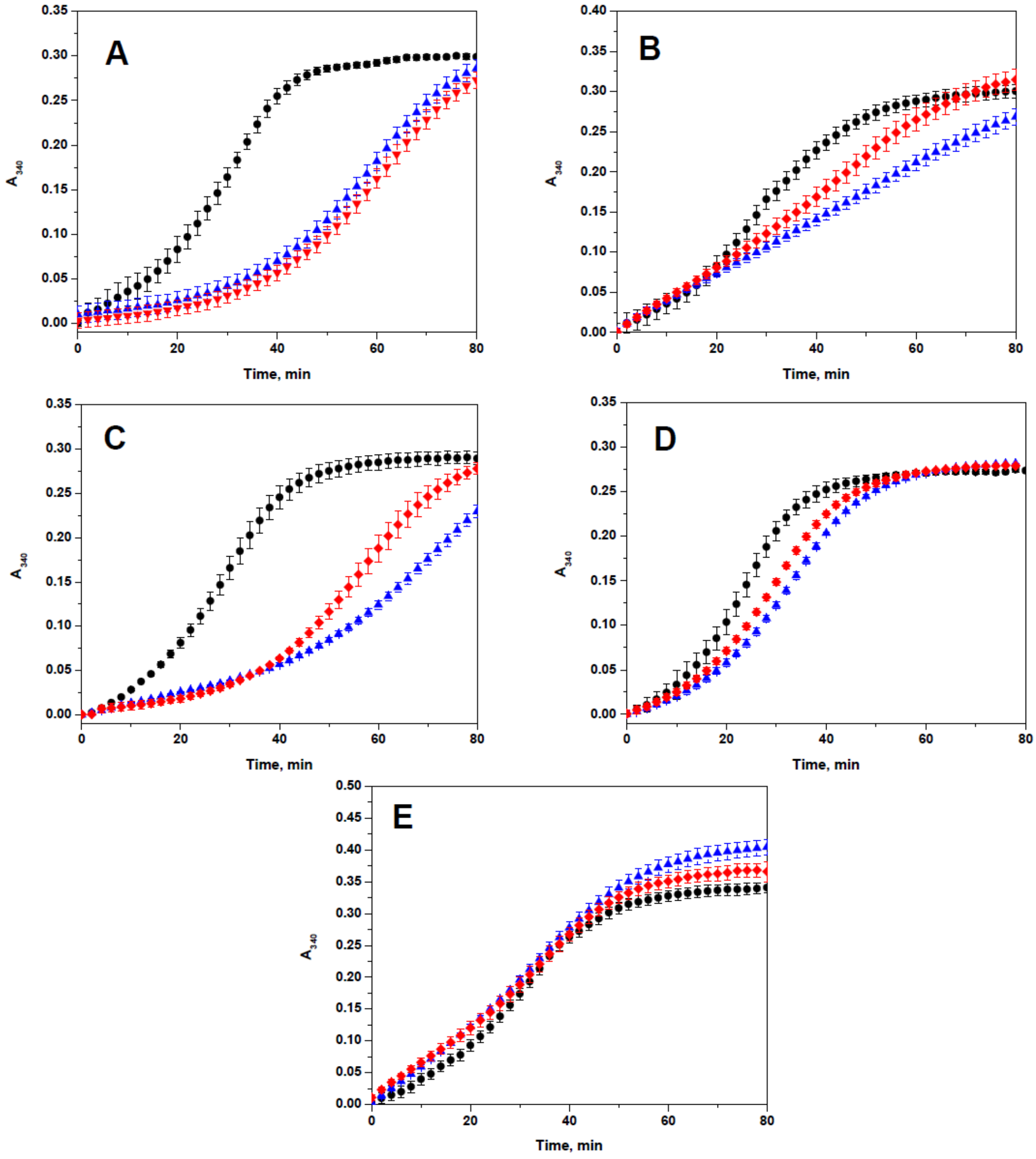

Supplementary Figure S6. Chaperone-like activity of HspB1 **(A)**, HspB4 **(B)**, HspB5 **(C)**, HspB6 **(D)** and HspB8 **(E)** with myosin subfragment-1 (S1) as a model substrate. Kinetics of aggregation of isolated S1 (black circles) and S1 in the presence of the wild type protein (blue triangles) or aggregation of S1 in the presence of corresponding R/A mutant (red diamonds) were recorded in no less than three independent experiments and data presented are mean with error bars corresponding to standard deviation. Weight ratios S1/sHsp was equal to 16 for HspB1, 20 for HspB4, 17 for HspB5, 10 HspB6, and 20 for HspB8.
